# The Constructive Black Queen hypothesis: new functions can evolve under conditions favouring gene loss

**DOI:** 10.1093/ismejo/wrae011

**Published:** 2024-01-23

**Authors:** Nobuto Takeuchi, Matthew S Fullmer, Danielle J Maddock, Anthony M Poole

**Affiliations:** School of Biological Sciences, University of Auckland, Auckland 1010, New Zealand; Universal Biology Institute, University of Tokyo, Tokyo 113-0033, Japan; Department of Biology, Faculty of Sciences, Kyushu University, Fukuoka 819-0395, Japan; School of Biological Sciences, University of Auckland, Auckland 1010, New Zealand; School of Biological Sciences, University of Auckland, Auckland 1010, New Zealand; School of Biological Sciences, University of Auckland, Auckland 1010, New Zealand

**Keywords:** Black Queen hypothesis, neofunctionalization, gene duplication, horizontal gene transfer, public goods, constructive neutral evolution, modelling

## Abstract

Duplication is a major route for the emergence of new gene functions. However, the emergence of new gene functions via this route may be reduced in prokaryotes, as redundant genes are often rapidly purged. In lineages with compact, streamlined genomes, it thus appears challenging for novel function to emerge via duplication and divergence. A further pressure contributing to gene loss occurs under Black Queen dynamics, as cheaters that lose the capacity to produce a public good can instead acquire it from neighbouring producers. We propose that Black Queen dynamics can favour the emergence of new function because, under an emerging Black Queen dynamic, there is high gene redundancy spread across a community of interacting cells. Using computational modelling, we demonstrate that new gene functions can emerge under Black Queen dynamics. This result holds even if there is deletion bias due to low duplication rates and selection against redundant gene copies resulting from the high cost associated with carrying a locus. However, when the public good production costs are high, Black Queen dynamics impede the fixation of new functions. Our results expand the mechanisms by which new gene functions can emerge in prokaryotic systems.

## Introduction

The evolution of new function is an ongoing process [1]. One route to new gene function is through duplication and divergence [1-5], a process which has resulted in numerous gene family expansions in eukaryotes [6, 7]. By contrast, the genomes of prokaryotes are frequently compacted and streamlined, owing to large population sizes [8, 9], and are subject to deletion bias [10-13]. Consistent with the compact architecture of many prokaryote genomes, there is evidence that pseudogenes and paralogs are underrepresented among streamlined prokaryote genomes [8, 14]. Pseudogenes may be rapidly cleared because mutations that result in protein truncations may yield toxic intermediates, which are strongly selected against [15]. Moreover, for periodically selected accessory genes, that serve a function in only some environments or conditions [16], there may be an advantage to their loss under conditions where the selection on them is relaxed [17], such as in an environment where their function is superfluous [14]. Thus, genome streamlining should impede the emergence of new gene functions. In the face of such loss, it appears that horizontal gene transfer (HGT) provides a major mechanism for the acquisition of a new function in prokaryotes [16, 18, 19].

Gene duplications in bacterial populations may nevertheless exist in steady state even where they incur a fitness cost [20], suggesting that deletion bias and fitness costs do not completely eliminate the raw materials for the emergence of new functions. It is also well documented that gene amplification can provide fitness benefits where increased gene dosage is advantageous [21-23], with amplification of weakly selected copies expediting the emergence of new function through duplication [1, 2].

It is also possible for nonessential genes to be retained because the avoidance of toxic intermediate formation may be bimodal: a locus that mutates to generate a toxic intermediate may either be lost very rapidly [15] or retained in the population in unmutated form despite no benefit [24] because both outcomes avoid the deleterious effect of toxic protein intermediates. In the latter instance, a functionally superfluous gene maintained by selection against such intermediates may provide the raw material for the exploration of new functional space. Nevertheless, current evidence suggests that, in comparison to eukaryotes, intragenomic locus duplication in prokaryotes appears to be a relatively minor route [19] for the emergence of new function.

We propose—and computationally test—a further mechanism for the emergence of new gene function that is robust to deletion bias and genome streamlining and which does not rely on intragenomic duplication events. In our model, new functions paradoxically emerge under Black Queen dynamics. The Black Queen Hypothesis [25] explains gene loss under conditions where goods cannot be monopolized. Within a microbial community where an essential public good is produced, cheaters that cease production of that good can evolve, leaving other community members stuck as producers (i.e. “Black Queens”) [25-27]. We hypothesize that Black Queen dynamics can in fact be constructive since the presence of a public good renders the responsible locus redundant in high copy number across a population. Provided public good production is maintained by a minority of community members, the loci in nonproducers are subject to relaxed selection. This creates the conditions where new function may evolve via the parallel exploration of mutational space. Our modelling results support our hypothesis that neofunctionalization can occur under Black Queen dynamics. We report that neofunctionalization emerges more rapidly via this route than via gene duplication alone, that neofunctionalization is favoured when public good cost is neither too low nor too high, and that neofunctionalization via this route can occur even where the cost of carrying a gene is high.

## Results

### The Constructive Black Queen hypothesis

**Figure 1:**
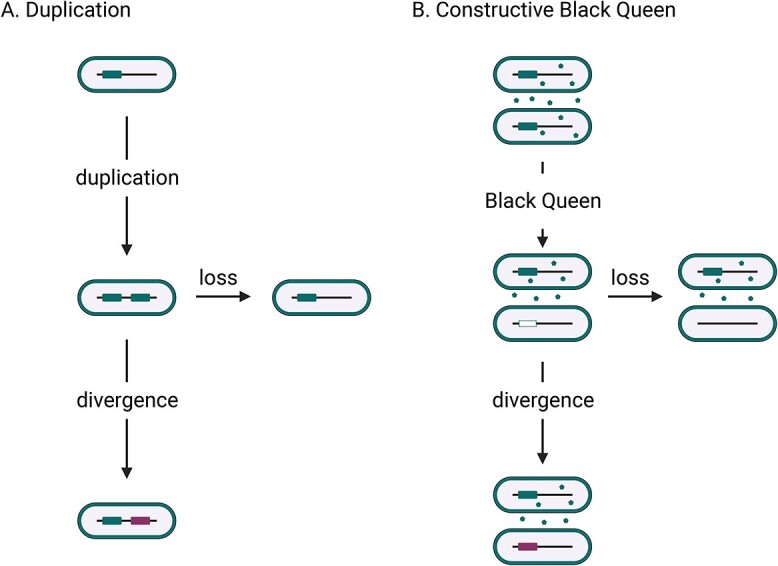
Neofunctionalization may occur via duplication and divergence or a cBQ; (A) a simple depiction of neofunctionalization via duplication; intragenomic duplication of a locus followed by mutation can lead to that locus being lost (loss pathway), else it may diverge, yielding some new function (divergence pathway, pink locus); (B) neofunctionalization is hypothesized to be possible under a Black Queen dynamic; the starting conditions are that all cells in a population carry a locus (blue) that codes for production of a good (blue dots); this public good cannot be monopolized (extracellular blue dots), which creates the conditions for the evolution of cheaters that do not contribute to public good production, yet derive benefit from its presence in the environment; cheaters may completely lose the public good locus, rendering them obligately dependent on producers (loss pathway); alternatively, it may be possible for the locus to diverge, giving rise to a new function (divergence pathway, pink locus); created with BioRender.com.

The basic premise of neofunctionalization is that, following a gene duplication event within an individual genome, the existence of two redundant copies enables one of those copies to diverge without detriment to the original function (Fig. 1A). It is thus possible for the diverged copy to acquire a new function [5], though loss of function through pseudogenization is the most likely fate.

We propose that a Black Queen dynamic creates conditions that increase the probability of neofunctionalization. Black Queen dynamics emerge where an essential or advantageous good is produced by the members of a population, but its public nature is such that the good cannot be monopolized [25, 27]. This in turn creates the conditions for cheaters to emerge, which derive benefit from the public good without contributing to its production. According to Black Queen theory, cheater phenotypes are manifested through the loss of genes required for public good production, with members of the population that have not lost this capacity becoming obligate producers [25, 26].

**Figure 2:**
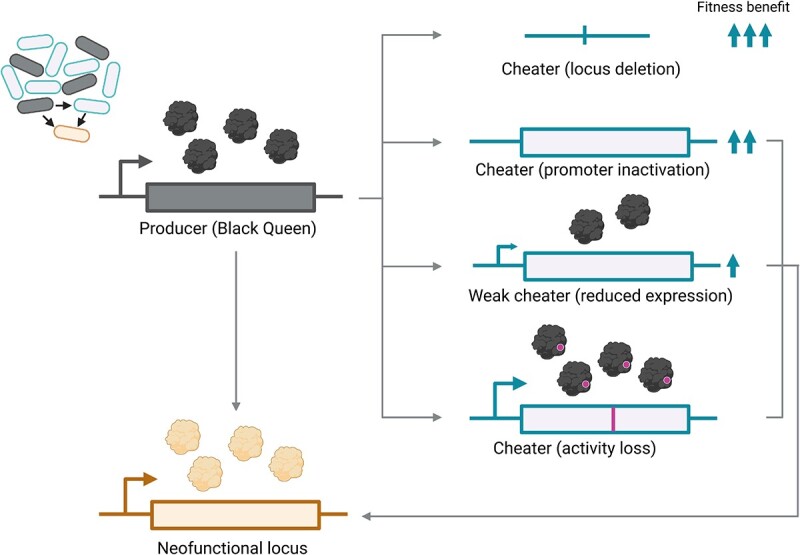
Multiple genotypes may be present in a population of cheaters during a Black Queen dynamic; a producer (Black Queen locus, black) can evolve new function (neofunctional locus, orange) because not all members of a community are required to produce a public good; direct mutation from one state to the other is possible in principle but unlikely; however, under a Black Queen dynamic, the population of cells diverges into producers and cheaters, with cheaters requiring proximity to producers to access the public good (cell population, top left); at the genetic level, multiple states may be present among cheaters (loci, right hand side) and may have varying fitness; locus deletion may be most selectively advantageous (no protein production, no locus maintenance, three upward arrows) but will not lead to neofunctionalization; however, three broad cheater genotype classes are, in principle, available for neofunctionalization; promoter inactivation retains the locus without expression; in this case, the cost of protein production is saved (two upward arrows), but the locus remains as raw material for neofunctionalization; a weak cheater may emerge through a promoter mutation that reduces but does not eliminate public good production; the cheater may nevertheless be selectively advantageous (one upward arrow) if it derives a greater portion of the public good from neighbouring cells than it contributes; neofunctionalization would render it an obligate cheater; a cheater phenotype may also arise through loss of activity; in this case, the mutation is selectively neutral; it is tolerated because loss of public good production has no detrimental effect, but the genotype is still able to produce (nonfunctional) protein; the expressed locus is under relaxed selection, so it is free to acquire new function; created with BioRender.com.

How might a Black Queen dynamic yield new function? We suggest that two key mechanisms may contribute. First, Black Queen dynamics generate genetic redundancy, which serves as the raw material for evolution of a new function. A Black Queen dynamic results in relaxed selection across the many copies of a locus within the population, as only a subset is required to support public good production. Importantly, and in contrast to gene duplication, this redundancy is intergenomic, not intragenomic, and is thus established without a duplication step (Fig. 1B). Second, at the molecular level, there are several ways in which a cheater phenotype may emerge. Complete deletion of the public good locus should eliminate all costs (expression and gene maintenance) and generate maximum benefit, provided there are public good producers present in the population. This genotype precludes neofunctionalization (Fig. 2). However, other genotype classes can lead to a cheater phenotype without locus loss, creating the prerequisite conditions for neofunctionalization under a Black Queen dynamic (Fig. 2). We consider three cases: (i) a mutation that eliminates gene expression without locus deletion would not bear any protein production costs, creating a cheater phenotype. (ii) A mutation that reduces gene expression but does not eliminate public good contributions could be considered a “weak cheater” as it derives more of the public good than it contributes. (iii) A mutation that inactivates the activity of a protein without eliminating protein production would be near neutral in that there is no cost to loss of function in the presence of public good producers, but neither is there a cost saving as protein is still produced.

A Black Queen dynamic thus has the potential to be “constructive” because the public good locus is under relaxed selection across many individuals within the population simultaneously. To test this hypothesis, we undertook computational modelling, with the aim of addressing the following questions: Are there conditions where a Black Queen dynamic can operate constructively? Are there conditions where a Black Queen dynamic is more likely to generate new function than gene duplication? Are there conditions where a Black Queen Dynamic hinders the evolution of new function?

**Figure 3:**
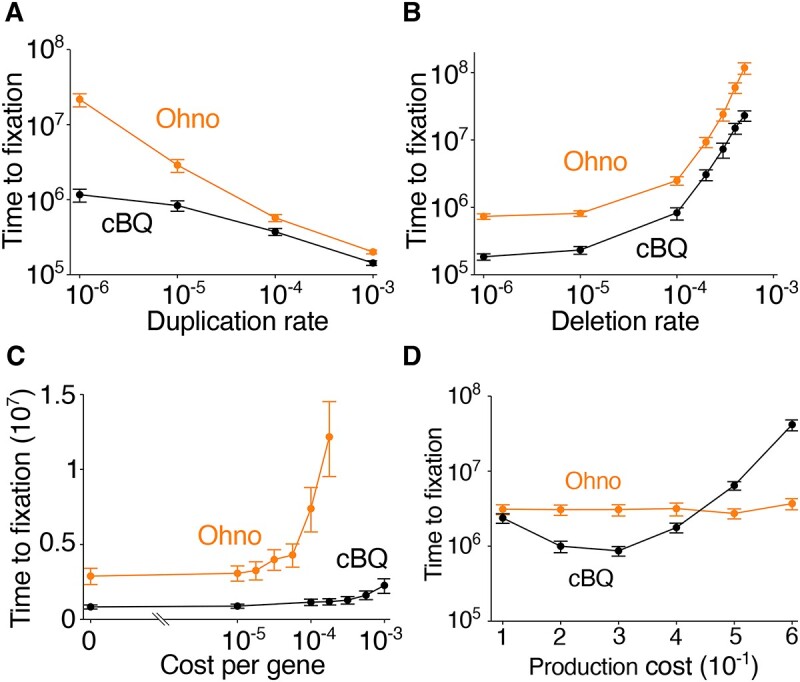
Black Queen dynamics can accelerate neofunctionalization; plots show the mean time (timesteps) for a new gene function to be fixed (present in >99% in total population) for a model with gene duplication alone (Ohno, orange) versus gene duplication plus a Black Queen dynamic (cBQ, black); duplication is implemented via “gene transfer” events (see Materials and methods); this enables the novel gene to also be acquired by producers in the cBQ model; error bars: 95% CI (100 replicate simulations and bootstrap with $N=10\ 000$); (A) Black Queen dynamics can accelerate neofunctionalization under deletion bias; when duplication rate is lower than gene deletion rate ($\delta ={10}^{-4}$), a cBQ substantially accelerates the evolution of new gene function relative to the Ohno model; at higher duplication rates, where deletion bias is eliminated, the difference between the two models is diminished; the cost of carrying a gene ($\gamma$) is zero, the cost of good production ($C$) is 0.5, and the benefit of neofunctional genes ($B$) is 0.07; for the cBQ model, the size of public-good sharing neighbourhood ($D$) is 3; (B) the acceleration of evolution by a cBQ dynamic diminishes as deletion rate increases; this contrasts with the result, shown in (A), that the relative acceleration increases as duplication rate decreases (see main text for explanation); duplication rate ($\chi$) is ${10}^{-5}$, $\gamma =0$, $C=0.5$, $B=0.07$ and, for the cBQ model, $D=3$; (C) in both models (Ohno, cBQ), the time taken to reach fixation increases as the cost of carrying genes increases, but this increase is much more rapid for the Ohno model; as the cost of carrying a gene increases, so does the strength of selection against gene redundancy; consequently, the time to fixation becomes very high under the Ohno model as intragenomic redundancy is selected against; under the cBQ model, this cost has less effect on the time to fixation (see main text for explanation); $\delta ={10}^{-4}$, $\chi ={10}^{-5}$, $C=0.5$, $B=0.07$, and for the cBQ model, $D=3$; (D) emergence of a cBQ depends on the cost of public good production; at moderate public good cost, a Black Queen dynamic accelerates fixation of a new gene, but when the cost of public good production is high, a Black Queen dynamic impedes fixation of a new gene; $\delta ={10}^{-4}$, $\chi ={10}^{-5}$, $\gamma =0$, $B=0.05$, and for the cBQ model, $D=5$.

### Black Queen dynamics can yield new gene function

To test whether a Black Queen dynamic can enhance neofunctionalization, we created two models, one that allows simple gene duplication (we call this model “Ohno”) and one that includes both gene duplication and public good sharing (we call this: “constructive Black Queen” (cBQ)). In our simulation, we have implemented gene duplication as an HGT process, meaning a locus may be both copied and transferred to another individual (see Materials and methods). Mutations can convert a gene (which is represented as a bit string of length four) from its original form (0000) to a neofunctional state (1111). In both models, the product of the original gene is essential (strong selection), whereas the neofunctional state is advantageous but nonessential. In the cBQ model, the product of the original gene is shared within a neighbourhood of the producer (public good), whereas in the Ohno model, it is exclusive to the producer (private good). The production gene (0000) can be inactivated by mutation but retained (any binary string except 0000 and 1111), or the entire locus may be deleted, yielding an obligate cheater. The model was implemented as a spatially extended individual-based model (see Materials and methods). All simulations start with a state in which all individuals carry one copy of the production gene.

To establish whether a Black Queen dynamic accelerates the rate of neofunctionalization, we ran simulations of both models and measured the time taken for a neofunctional gene (1111) to be fixed for a range of duplication rates. A gene is considered fixed when its frequency exceeds 99% in the total population, including both producers and cheaters (note both models permit acquisition by HGT). Our results indicate that the fixation time in the Ohno model increases as duplication rate decreases (Fig. 3A), a result that makes intuitive sense because duplication is essential for neofunctionalization in this model. By contrast, the fixation time in the cBQ model levels off as the duplication rate decreases (Fig. 3A), indicating that a Black Queen dynamic substantially accelerates the fixation of new functions under conditions where duplication rate is lower than the gene deletion rate (i.e. reflecting a deletion bias). This result can be understood from the fact that cheaters that bring genetic redundancy to the system are generated by mutation and are favoured by natural selection (Fig. S1) so that their existence is independent of gene duplication. At higher rates of duplication (≥10^−4^), the two models display similar rates of fixation (Fig. 3A), which is expected because duplication is the predominant pathway for neofunctionalization for this parameter range.

We examined how deletion affects the evolution of a new function by running simulations for a range of deletion rates. Our results show that the fixation time of a neofunctional gene in the cBQ model is consistently shorter than in the Ohno model for a wide range of deletion rates, indicating a several-fold acceleration of fixation in the cBQ model (Fig. 3B). However, the relative magnitude of the acceleration decreases as the deletion bias increases. This is in contrast with the result shown in Fig. 3A, where the fold difference in fixation time increases as the duplication rate decreases (note that a decreasing duplication rate also implies an increasing deletion bias). These contrasting results can be understood from the fact that, for a Black Queen dynamic, duplication is not essential for the production of genetic redundancy, whereas deletion directly reduces this redundancy. Therefore, the acceleration by Black Queen dynamics is more robust to low duplication rates than to high deletion rates.

In the simulations described above, we permitted deletion bias, but we did not include the cost associated with carrying a gene. The original Black Queen hypothesis describes a population splitting into individuals carrying the public good locus and those that have lost it [25]. The latter may have an advantage relative to other forms of cheater if there is a cost to carrying a gene. We therefore repeated our simulations, but this time added a cost for carrying a gene locus. One possible outcome is that cheaters that lack the locus entirely would out-compete other forms of cheater, potentially impeding neofunctionalization via a Black Queen dynamic (Fig. 2). Moreover, gene duplication events would also be disadvantageous because carrying a duplicate copy comes at a cost, which can hinder neofunctionalization through the Ohno pathway. Our result shows that, as the cost of carrying a gene increases, the rate of fixation of a new function under the Ohno model rapidly declines as expected, whereas this effect is minor in the cBQ model even as the per-gene cost becomes high (Fig. 3C). This suggests that, under conditions that favour genomic streamlining, a Black Queen dynamic can also be constructive even though cheaters that have completely lost the locus should be at a short-term advantage.

**Figure 4:**
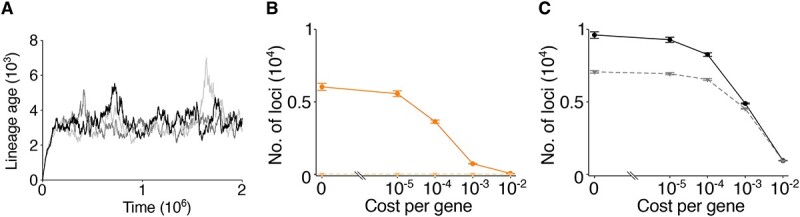
Genetic redundancy is robust against gene cost in cBQ model; (A) plot shows the average age of cheater lineages as a function of time in the cBQ model; age count starts when a cheater arises from a producer through mutation or deletion and increases by one when a cheater reproduces (see Materials and methods); cost per gene ($\gamma$) is set to ${10}^{-4}$, and the other parameters are the same as in Fig. 3C, except that the benefit of neofunctional genes ($B$) is set to zero so that the age of lineages represents a situation before the evolution of a new gene function; results from three replicate simulations are shown; the plot shows that the mean age of lineages is ~3000 generations; if cost for carrying a gene is denoted by $\gamma$, selection induced by this cost has a timescale of approximately ${\gamma}^{-1}$ generations; thus, the plot indicates that the timescale of selection induced by cost for carrying a gene is slower than the turnover of cheater lineages if $\gamma <{10}^{-3}$; (B) the mean total number of “mutable” loci across both producer and cheater populations (circles, solid line) or only the cheater population (triangles, broken line), as a function of cost for carrying a locus in the Ohno model; (C) the mean total number of “mutable” loci across both producer and cheater populations (circles, solid line) or only the cheater population (triangles, broken line), as a function of cost for carrying a locus in the cBQ model; in panels (B) and (C), mutable loci are defined as those that can mutate without reducing the fitness of the carriers; the number of mutable loci quantifies the degree of genetic redundancy; the plots show that the number of mutable loci decreases as the cost increases in both models, but this decrease is slower in the cBQ model, indicating that selection against genetic redundancy is buffered; the parameters are the same as in (A); each simulation was run for $5.6\times{10}^6$ time steps, and the number of loci was averaged over time with the first ${10}^6$ time steps discarded; error bars: 95% CI (100 replicate simulations and bootstrap with $N=10\ 000$).

To understand why the cBQ model is robust to the high cost for carrying genes, we measured the age of cheater lineages, where age count starts when a cheater arises from a producer through mutation or deletion and increases by one when a cheater reproduces (see Materials and methods). If cheater lineages persist indefinitely, their age is expected to increase without bound as time increases. Our results, however, show that the average age of cheater lineages levels off at ~3000 generations (Fig. 4A). This means that the cheater population is constantly replaced through the ongoing emergence of cheaters from producers by mutation or deletion. This rapid turnover likely occurs because recently derived cheaters have a selective advantage relative to those derived earlier. This advantage stems from the fact that recently derived cheaters are more likely to be spatially proximate to producers. Thus, the advantage is independent of whether cheaters arise through mutation or deletion. This advantage can explain why the cBQ model is robust to cost for carrying genes. In our model, a cheater can retain genetic redundancy and can thus evolve new function if its production locus has not been deleted. The abundance of such cheaters and, hence, genetic redundancy in the system are expected to be only weakly dependent on cost for carrying genes because selection against genetic redundancy induced by this cost is buffered by the rapid turnover of the cheater population caused by newly arising cheaters, which have a selective advantage due to spatial proximity to producers even if their production locus has not been deleted. By contrast, the Ohno model has no such mechanism to buffer this selection against redundant loci.

To test whether selection against genetic redundancy is buffered in the cBQ model, we measured the total number of redundant loci across a population as a function of cost for carrying genes, in both Ohno and cBQ models. We quantified genetic redundancy by counting the total number of loci, across a population, that can mutate without reducing the fitness of the carrier (such loci include mutated production loci in cheaters and duplicated production loci in producers). Our results show that genetic redundancy decreases as the cost for carrying genes increases but that this occurs more rapidly in the Ohno model than in the cBQ model (Fig. 4B and C). Moreover, when this cost is high, most of the redundant loci are carried by cheaters in the cBQ model (Fig. 4C). This suggests that selection against genetic redundancy is indeed buffered in the cBQ model.

We next asked whether there are conditions where the Black Queen dynamic could impede the evolution of new function. To this end, we measured the time to fixation of new gene function under increasing public good production costs (Fig. 3D). Our results show that, when the production cost is low (${10}^{-1}$), the difference between the Black Queen and Ohno models is small. At an intermediate production cost (e.g. $3\times{10}^{-1}$), the Black Queen dynamic accelerates the evolution of new function. However, as the cost of public good production increases, the difference is again dimished. Finally, when production cost is high (e.g. $5\times{10}^{-1}$), the Black Queen dynamic actually impedes the evolution of new functions (Fig. 3D). These results indicate that a Black Queen dynamic can either accelerate or decelerate the evolution of novel function depending on the production cost.

**Figure 5:**
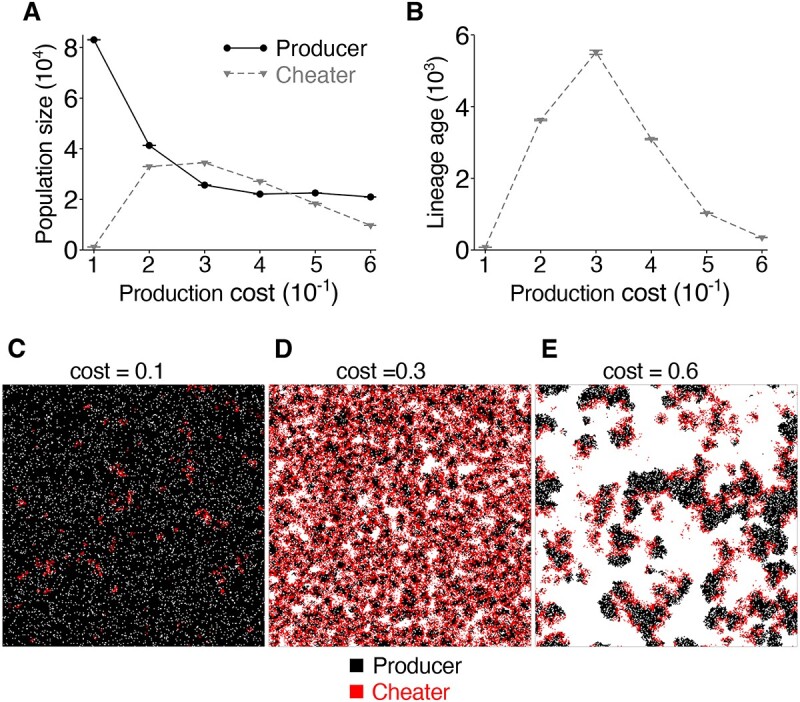
Emergence of a cBQ depends on the population size and lineage longevity of cheaters; (A) the plot shows the population sizes of producers (circles, solid line) and cheaters (triangles, broken line) averaged over time as a function of public good production cost for the cBQ model; the population size of cheaters is high at moderate public good cost, but low when this cost is low or high; error bars: 95% CI (100 replicate simulations and bootstrap with $N=10\ 000$); the parameters are the same as in Fig. 3D, except that the benefit of neofunctional genes ($B$) is set to zero so that the results represent a situation before the evolution of a new gene function (the same parameters are used in panels (B)–(E); (B) the plot shows the average age of parasite lineages as a function of public good production cost; at intermediate public good production cost ($3\times{10}^{-1}$), the lifespan of cheaters is high $(\sim 6000$ generations), leaving ample time for the exploration of sequence space and thus neofunctionalization is expedited; at high public good production cost ($\ge 5\times{10}^{-1}$), the lifespan of cheaters is low ($<2000$ generations); cheaters are thus rapidly turned over, reducing the opportunity for mutation events to yield new function; lower panels: snapshots of simulations depicting spatial distributions of cheaters (red) and producers (black) on the model grid for different public good costs (denoted by $C$); (C) at low cost ($C={10}^{-1}$), cheaters are rare while producers are abundant; this reduces the number of loci at which a novel gene function may emerge; consequently, the Black Queen dynamic is only very weakly constructive; (D) at intermediate cost ($C=0.3$), cheaters become abundant, and there are thus many more opportunities for the evolution of a new gene function; (E) at high cost ($C=0.6$), cheaters induce travelling wave patterns, which slows down the evolution of novel functions.

We sought to establish exactly what is happening at low, medium, and high public good costs. When the cost of producing a public good is low, we find that cheaters are rare (Fig. 5A). This is because converting from a producer to a cheater is not strongly advantageous since the cost of production is low. Fewer cheaters mean there are fewer opportunities for a Black Queen to operate constructively, explaining the similarity of fixation rate in the cBQ and Ohno models (Fig. 3D). At both intermediate and high production costs, cheaters are at an advantage and become relatively abundant (Fig. 5A); nevertheless, the Black Queen dynamic inhibits the evolution of new function when production costs are high (Fig. 3D). We hypothesized that the difference in neofunctionalization at intermediate and high production costs is due to differences in the turnover of the cheater population. If turnover of the cheater population is high, there can be frequent emergence of cheater lineages, but few are sufficiently long-lived to evolve new function. By contrast, long-lived cheater lineages should have a greater probability of the evolving novel gene function. We therefore examined the age of cheater lineages at intermediate and high public good costs. We found that, when public good production costs are intermediate ($3\times{10}^{-1}$), the average age is ~6000 generations (Fig. 5B), whereas at high cost ($5\times{10}^{-1}$), the average age of cheaters is far below 2000 (Fig. 5B). This shortened lifespan of cheater lineages indicates that new cheaters are emerging rapidly (large advantage) but are rapidly turned over, shortening the paths explored by cheaters in genotype space. A critical difference at low and high public good costs is that, at high public good cost, parasites are at greater risk of extinction because they can also cause the localized extinction of hosts (Fig. 5C–E), as indicated by a smaller total population size at high public good cost (Fig. 5A). This is because parasites are at a strong advantage over neighbouring hosts, the latter bearing the cost of production. Outcompeting hosts drives them locally extinct and results in the subsequent extinction of neighbouring parasites, which creates a rolling wave effect with a high parasite turnover (Fig. 5E). Taken together, the above results indicate that the sweet spot for evolution of new functions via a cBQ is found where a public good has a moderate production cost (Fig. 3D), the longevity of cheater lineages is great, and the frequency of cheaters is high (Fig. 5A and B).

## Discussion

The Black Queen hypothesis, as originally conceived, focused on the emergence of cheaters that had lost the capacity to synthesize a public good [25]. Black Queen dynamics have since been shown to evolve in laboratory conditions [27], and it has been noted that, where there are multiple public good loci, the probability of all public good genes being present in one lineage with other lineages being cheaters is low, meaning that ecological interdependencies may evolve [26]. In the present work, we have presented and tested the hypothesis that there should be conditions where a Black Queen dynamic is constructive, enhancing the emergence of new gene function (neofunctionalization) despite such a dynamic being characterized by loss of function.

Our modelling results show that there are conditions wherein a Black Queen dynamic can enhance neofunctionalization over a model with simple duplication alone (Fig. 3). We find that this occurs even when genome streamlining is favoured by low duplication rates or the high cost of carrying a gene. We also find that a Black Queen operating constructively depends on the cost of public good production (Fig. 3D). When this cost is very low, there is no difference between traditional gene duplication alone and a model that also includes a Black Queen. This suggests that the Black Queen has no detectable impact on neofunctionalization under these conditions (and may even have a slightly inhibitory effect, Fig. 3D). At intermediate production cost, a Black Queen dynamic is constructive and significantly accelerates the fixation of new function. However, when the cost of public good production is high, this tendency reverses and, at very high production costs, Black Queen dynamics are strongly inhibitory to neofunctionalization. Together, our results indicate that the idea of a cBQ is plausible and that there are conditions where a cBQ is much more likely to generate a new function than gene duplication alone.

Our proposed mechanism for neofunctionalization differs substantially from existing models for the evolution of new function [7, 19, 28-30] for several reasons. First, it does not involve an intragenomic duplication step, so it is distinct from neo- or subfunctionalization of paralogous gene copies [1, 2, 4]. Second, it is distinct from the creation of intragenomic redundancy following HGT, where xenologous gene copies may functionally diverge [19], and from the *de novo* emergence of new gene function from a region overlapping an existing coding region (overprinting) [28, 30, 31], or from random sequences [29]. Finally, it precludes subfunctionalization [32] as an outcome because the redundancy is between gene copies in different cells. Thus, there is no opportunity for a function to be shared between diverged copies.

The cBQ thus adds another mechanism to the arsenal of mechanisms that contribute to neofunctionalization. Importantly, and in contrast to other mechanisms discussed above, this does not involve a physical gene duplication, either via the creation of an additional copy of a genetic locus through intragenomic recombination, or via the introduction of a functionally redundant xenolog via HGT. Instead, it is wholly intergenomic. Once a new gene function emerges, it may of course spread via HGT, but, in contrast to neofunctionalization following HGT [19], a new function generated via a cBQ has already arisen, so HGT spreads an established function rather than the raw material for neofunctionalization. This helps to explain how bacteria with strong deletion biases or streamlined genomes may nevertheless evolve new functions and adds to the mechanisms for evolution of new functions in prokaryotes.

Our results indicate that Black Queen loci may be hotspots for the exploration of a new gene function. However, it is important to note that our results suggest that not all such loci would be constructive, with some potentially being inhibitory to neofunctionalization. This appears to be the case for public goods that are particularly costly to produce. However, even under very strong deletion bias, the Black Queen can be constructive, suggesting that under such conditions, neofunctionalization via duplication or gene gain is exceedingly rare, so cBQs may have a proportionately greater role in the evolution of new function.

While Black Queen dynamics are based on loss of function, our results suggest that they can provide an important mechanism for neofunctionalization in species subject to genome streamlining. If correct, this mechanism may lessen the impact of the loss of adaptive capacity under streamlining [14] and may provide an additional mechanism through which functional diversity evolves among streamlined lineages. The cBQ demonstrates that, even under the extremes of genome streamlining, where population sizes and strength of selection are such that deletion bias and selection against superfluous genetic material occurs, there is still the prospect of genetic innovation; constructive evolution can emerge from the crucible of selection for gene loss. It is noteworthy that single-cell sequencing of uncultured marine bacteria has identified genomically streamlined oligotrophs from the phyla Verrucomicrobia and Bacteriodetes with elevated levels of extracellular, outer membrane and multilocation proteins [33], exactly the kinds of gene function that might be associated with Black Queen dynamics. It will be interesting to establish whether the cBQ dynamics we describe here contribute to such diversification and expansion of gene function.

While diversification of function via the cBQ is restricted to public goods systems, the resulting products need not be. Nevertheless, new functions that emerge via this route may in turn be public goods (e.g. extracellular hydrolase gene loci undergoing a Black Queen dynamic may yield a new extracellular hydrolase function), so cBQ dynamics may provide a mechanism for the further expansion of public good systems; one public good may beget another. Together with the observations that HGT of public good genes, including those encoding secreted proteins, can drive bacterial cooperation [34, 35], the dynamics we describe add to the diversification of new and more complex microbial community networks, as envisaged under a “strong” Black Queen [26], which notes that, where there are multiple public goods, it is unlikely that production of all goods are constrained to one lineage alone. It is tempting to speculate that the formation of community interactions via Black Queen-based gene loss, expansion of cooperative networks, and metabolic diversification via cBQ dynamics go hand in hand.

## Materials and methods

### Model

Our model is an agent-based model of bacterial genome evolution. To incorporate local interactions, the model is implemented as a square grid on which bacteria are spatially distributed. Each square on the grid (pixel, for short) can have at most one individual bacterium or be empty. The population size of bacteria is thus bounded above by the total number of pixels (denoted by $N$). The dimension of the grid is set to 300-by-300 pixels unless otherwise stated (thus, $N=90\ 000$). The boundary condition is toroidal (i.e. wrapped around) to remove boundary effects.

The genome of a bacterium is assumed to consist of one or more genes. A gene is modelled as a bit string of length four: 0000 is referred to as a good-production gene; 1111 is referred to as a neofunctional gene; and any other bit string is referred to as an inactive gene. The presence or absence of different genes affects the reproduction rate of bacteria, as described next.

The reproduction rate of bacterium $i$ (denoted by ${r}_i$) is defined as


$$ {r}_i=\max \left(-{c}_i+{b}_i-{\gamma g}_i+{p}_i,0\right), $$

where ${c}_i$ is the cost of good production (${c}_i=C$ if bacterium $i$ carries at least one good-production gene; otherwise, ${c}_i=0$), ${b}_i$ is the benefit of carrying neofunctional genes (${b}_i=B$, if bacterium $i$ carries at least one neofunctional gene; otherwise, ${b}_i=0$), $\gamma$ is the per-gene cost of carrying genes, ${g}_i$ is the number of genes carried by bacterium $i$, and ${p}_j$ is the benefit of obtaining good produced by another bacterium or bacterium $i$ itself (${p}_i=P$, if bacterium $i$ obtains good; otherwise, ${p}_i=0$; $P=1$ unless otherwise stated). Whether bacterium $i$ obtains good or not is probabilistically determined, as follows. One pixel is randomly chosen with an equal probability from a region of $D$-by-$D$ pixels centred around bacterium $i$, including the pixel in which bacterium $i$ resides. If the chosen pixel contains a bacterium carrying good-production genes, bacterium $i$ receives good (${p}_i=P$); otherwise, it does not (${p}_i=0$). If $D>1$, good can be shared between multiple bacteria (public good), so the model allows for Black Queen dynamics (cBQ model). By contrast, if $D=1$, the good is exclusively used by the producer (private good). In this case, the model does not allow for Black Queen dynamics (Ohno model). Note that obtaining good is essential for bacterial reproduction because the reproduction rate is zero (${r}_i=0$) if a bacterium neither obtains good (${p}_i=0$) nor has neofunctional genes (${b}_i=0$).

While different bacteria reproduce at different rates as described above, all bacteria are assumed to die at an equal rate (denoted by $d$) for simplicity. The value of $d$ was set to 0.05.

In each time step, the state of the grid is updated by repeating the following algorithm $N/\alpha$ times, where $\alpha$ is a time scaling parameter (explained in the next paragraph). One pixel is randomly chosen from the grid with an equal chance. If the chosen pixel contains a bacterium, the bacterium dies with probability $\alpha d$ ($d=0.01$ unless otherwise stated). Alternatively, if the chosen pixel is empty, a new bacterium can be created in it, as follows. First, another pixel is randomly chosen from the eight pixels neighbouring the pixel chosen first (i.e. the Moore neighbourhood excluding the central pixel) with an equal chance. If the chosen neighbouring pixel contains a bacterium (denoted by $i$), bacterium $i$ can reproduce with probability $\alpha{r}_i$, creating a new bacterium in the empty pixel, which is chosen first.

The scaling parameter $\alpha$ introduced above is set such that $\alpha d$ and $\alpha{r}_i$ are smaller than unity so that they can be considered as probabilities. The specific value of $\alpha$ does not matter for the outcome of simulations: one time step in the model constitutes the repetition of the above algorithm $N/\alpha$ times so that $\alpha$ cancels out.

When a new bacterium is created, it inherits the genome of its parent with potential modifications, as follows. Genes can undergo point mutations (bit flipping) with probability $\mu$ per bit ($\mu ={10}^{-4}$). Genes can also be deleted from the genome with probability $\delta$ per gene ($\delta ={10}^{-4}$). The bacterium can also gain a new gene through gene duplication or HGT, as follows. When a new bacterium is created, one pixel is randomly chosen from a region of three-by-three pixels centred around the new bacterium (i.e. the Moore neighbourhood) with an equal probability. If the chosen pixel contains a bacterium, one gene is randomly chosen from this bacterium with an equal chance (if the pixel is empty, nothing occurs). A copy of the chosen gene is added to the genome of the new bacterium with probability $\chi$. This process can be regarded as gene duplication if the receiver is identical to the donor, or otherwise, as HGT. However, for brevity, $\chi$ is referred to as the duplication rate. Note that results are equivalent if the gene duplication is implemented as an intragenomic gene duplication process (Fig. S2).

### Measurement of the age of lineages

To measure the age of lineages in the models, every individual bacterium is attached with an age counter. The age counter of every bacterium is set to zero at the beginning of a simulation. When a new bacterium is produced, its age counter is set to one plus the age count of its parent if both parent and offspring are either producers or cheaters. However, its age counter is set to zero if it is a cheater (or producer), but its parent is a producer (or cheater, respectively). A bacterium is a producer if it carries at least one copy of the production gene; otherwise, it is a cheater.

## Supplementary Material

3supp_Takeuchi_BQ_231213_wrae011

## Data Availability

The source codes implementing the models used in this study are available in the Figshare repository: https://doi.org/10.17608/k6.auckland.24934764.
