# Supplementary material for: The Constructive Black Queen hypothesis: new functions can evolve under conditions favouring gene loss
Source: ISME J. 2024 Jan 23;18(1):wrae011. doi: 10.1093/ismejo/wrae011 (PMC10942775; doi:10.1093/ismejo/wrae011)
Supplement: 3supp_Takeuchi_BQ_231213_wrae011 [file 3supp_takeuchi_bq_231213_wrae011.pdf]

Supplementary Information: The Constructive Black Queen hypothesis: new functions can evolve under conditions favouring gene loss.

Nobuto Takeuchi[1,2,3], Matthew S. Fullmer[1+], Danielle J. Maddock[1+], Anthony M. Poole[1*]

[1]School of Biological Sciences, University of Auckland, Auckland, New Zealand

[2]Universal Biology Institute, University of Tokyo, Tokyo, Japan

[3]Department of Biology, Faculty of Sciences, Kyushu University, Fukuoka, Japan

+These authors contributed equally

*Correspondence: a.poole@auckland.ac.nz

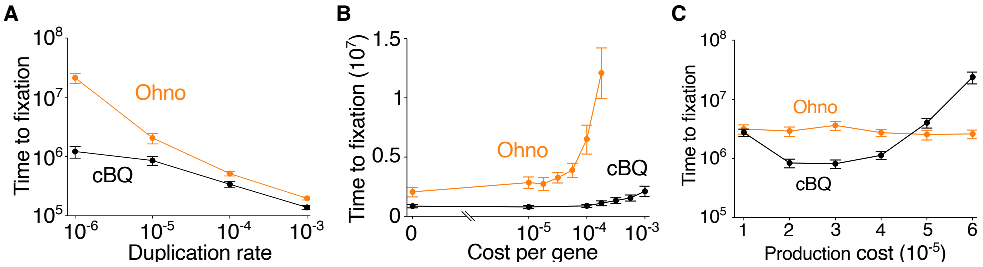

Figure S1. Black Queen dynamics can accelerate neofunctionalization when duplication is modelled as an intragenomic gene duplication process. Plots show the mean time (timesteps) for a new gene function to be fixed for a model with gene duplication alone (Ohno, orange) versus gene duplication plus a Black Queen dynamic (cBQ, black). Duplication is implemented via intragenomic gene duplication events. The lack of horizontal gene transfer means that a novel gene function that evolves in one member of the population (either a cheater or producer) cannot be acquired by other members of the population. Consequently, a novel gene function evolving in a cheater will be limited in its capacity to reach fixation because there is also selection favouring cheaters that are recently derived from—and, thus, spatially proximate to—producers, which lack a novel gene. It is therefore improbable for a cheater with a novel gene function to reach >99% frequency. Thus, to capture the difference between the Ohno and cBQ models, a novel gene was regarded as being 'fixed' when it was present in >20% (instead of 99%) of the cheater or producer population. Error bars: 95% CI (100 replicate simulations and bootstrap with $N = 10{,}000$). **A.** Black Queen dynamics can accelerate neofunctionalization under deletion bias. The cost of carrying a gene (γ) is zero, the cost of good production (C) is 0.5, and the benefit of neofunctional genes (B) is 0.07. For the cBQ model, the size of public-good sharing neighbourhood (D) is 3. Gene duplication rate (χ) varies, while gene deletion rate ($\delta = 10^{-4}$) is kept constant. As duplication rate decreases, this generates a deletion bias. **B.** In both models (Ohno, cBQ), the time taken to reach fixation increases as the cost of carrying genes increases, but the increase is much more rapid for the Ohno model. $\delta = 10^{-4}$, $\chi = 10^{-5}$, $C = 0.5$, and $B = 0.07$. For the cBQ model, $D = 3$. **C.** Emergence of a constructive Black Queen depends on the cost of public good production (C). At moderate public good cost, a Black Queen dynamic accelerates fixation of a new gene. $\delta = 10^{-4}$, $\chi = 10^{-5}$, $\gamma = 0$, and $B = 0.05$. For the cBQ model, $D = 5$.

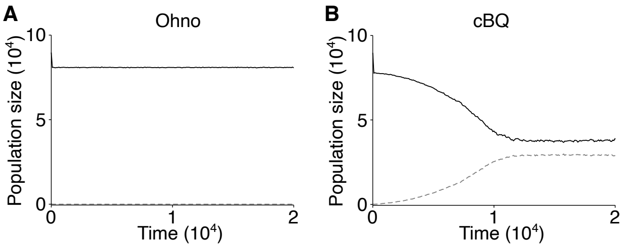

Figure S2. Cheaters are favoured by natural selection in the cBQ model. The plots show the population sizes of producers (black, solid lines) and cheaters (grey, dashed lines) in the Ohno (**A**) and cBQ (**B**) models. Simulations start with a state in which an entire grid is occupied by individuals carrying one copy of the production gene (producers), except for a circular region of radius 10 pixels that is occupied by individuals carrying one mutated production locus (cheaters). In the Ohno model, cheaters cannot invade as there is no public good production whereas, in the cBQ model, cheaters can invade and stably coexist with producers, indicating that cheaters are favoured by selection when rare. The parameters are the same as in Figure 3A, except that mutation rate (μ) and deletion rate (δ) are set to zero so we can examine whether cheaters can invade when no new cheaters are produced.
